# JCOG0911 INTEGRA study: a randomized screening phase II trial of interferonβ plus temozolomide in comparison with temozolomide alone for newly diagnosed glioblastoma

**DOI:** 10.1007/s11060-018-2831-7

**Published:** 2018-03-20

**Authors:** Toshihiko Wakabayashi, Atsushi Natsume, Junki Mizusawa, Hiroshi Katayama, Haruhiko Fukuda, Minako Sumi, Ryo Nishikawa, Yoshitaka Narita, Yoshihiro Muragaki, Takashi Maruyama, Tamio Ito, Takaaki Beppu, Hideo Nakamura, Takamasa Kayama, Shinya Sato, Motoo Nagane, Kazuhiko Mishima, Yoko Nakasu, Kaoru Kurisu, Fumiyuki Yamasaki, Kazuhiko Sugiyama, Takanori Onishi, Yasuo Iwadate, Mizuhiko Terasaki, Hiroyuki Kobayashi, Akira Matsumura, Eiichi Ishikawa, Hikaru Sasaki, Akitake Mukasa, Takayuki Matsuo, Hirofumi Hirano, Toshihiro Kumabe, Nobusada Shinoura, Naoya Hashimoto, Tomokazu Aoki, Akio Asai, Tatsuya Abe, Atsuo Yoshino, Yoshiki Arakawa, Kenichiro Asano, Koji Yoshimoto, Soichiro Shibui, Members of Japan Clinical Oncology Group Brain Tumor Study Group (JCOG-BTSG)

**Affiliations:** 1 Department of Neurosurgery, Nagoya University Graduate School of Medicine, Nagoya, Japan; 2 JCOG (Japan Clinical Oncology Group) Data Center/Operations Office, National Cancer Center Hospital, Tokyo, Japan; 3 Radiation Oncology Department, Cancer Institute Hospital, Tokyo, Japan; 4 Department of Neuro-Oncology/Neurosurgery, Saitama Medical University International Medical Center, Saitama, Japan; 5 Department of Neurosurgery and Neuro-Oncology, National Cancer Center Hospital, Tokyo, Japan; 6 Department of Neurosurgery, Tokyo Women’s Medical University, Tokyo, Japan; 7 Department of Neurosurgery, Nakamura Memorial Hospital, Sapporo, Japan; 8 Department of Neurosurgery, Iwate Medical University, Morioka, Japan; 9 Department of Neurosurgery, Kumamoto University Graduate School of Medicine, Kumamoto, Japan; 10 Department of Neurosurgery, Yamagata University Graduate School of Medicine, Yamagata, Japan; 11 Department of Neurosurgery, Kyorin University Faculty of Medicine, Mitaka, Japan; 12 Department of Neurosurgery, Shizuoka Cancer Center, Shizuoka, Japan; 13 Department of Neurosurgery, Hiroshima University, Graduate School of Biomedical and Health Sciences, Hiroshima, Japan; 14 Department of Clinical Oncology & Neuro-oncology Program, Hiroshima University Hospital, Hiroshima, Japan; 15 Department of Neurosurgery, Ehime University Graduate School of Medicine, Ehime, Japan; 16 Department of Neurosurgery, Chiba University Graduate School of Medicine, Chiba, Japan; 17 Department of Neurosurgery, Kurume University Graduate School of Medicine, Kurume, Japan; 18 Department of Neurosurgery, Hokkaido University Graduate School of Medicine, Sapporo, Japan; 19 Department of Neurosurgery, Faculty of Medicine, University of Tsukuba, Tsukuba, Japan; 20 Department of Neurosurgery, Keio University School of Medicine, Tokyo, Japan; 21 Department of Neurosurgery, The University of Tokyo Hospital, Tokyo, Japan; 22 Department of Neurosurgery, Nagasaki University Graduate School of Medicine, Nagasaki, Japan; 23 Department of Neurosurgery, Kagoshima University Graduate School of Medical and Dental Sciences, Kagoshima, Japan; 24 Department of Neurosurgery, Tohoku University Graduate School of Medicine, Sendai, Japan; 25 Present Address: Department of Neurosurgery, Kitasato University School of Medicine, Kanagawa, Japan; 26 Department of Neurosurgery, Tokyo Metropolitan Cancer and Infectious Disease Center Komagome Hospital, Tokyo, Japan; 27 Department of Neurosurgery, Osaka University Graduate School of Medicine, Suita, Japan; 28 Department of Neurosurgery, Kitano Hospital, Osaka, Japan; 29 Department of Neurosurgery, Kansai Medical University, Osaka, Japan; 30 Department of Neurosurgery, Oita University Faculty of Medicine, Yufu, Japan; 31 Department of Neurological Surgery, Nihon University Graduate School of Medicine, Tokyo, Japan; 32 Department of Neurosurgery, Kyoto University Graduate School of Medicine, Kyoto, Japan; 33 Department of Neurosurgery, Hirosaki University Graduate School of Medicine, Hirosaki, Japan; 34 Department of Neurosurgery, Graduate School of Medical Sciences, Kyushu University, Fukuoka, Japan

**Keywords:** Glioblastoma, Interferon-beta, Temozolomide, MGMT, RCT

## Abstract

**Purpose:**

This study explored the superiority of temozolomide (TMZ) + interferonβ (IFNβ) to standard TMZ as treatment for newly diagnosed glioblastoma (GBM) via randomized phase II screening design.

**Experimental design:**

Eligibility criteria included histologically proven GBM, with 50% of the tumor located in supratentorial areas, without involvement of the optic, olfactory nerves, and pituitary gland and without multiple lesions and dissemination. Patients in the TMZ + radiotherapy (RT) arm received RT (2.0 Gy/fr/day, 30 fr) with TMZ (75 mg/m^2^, daily) followed by TMZ maintenance (100–200 mg/m^2^/day, days 1–5, every 4 weeks) for 2 years. Patients in the TMZ + IFNβ + RT arm intravenously received IFNβ (3 MU/body, alternative days during RT and day 1, every 4 weeks during maintenance period) and TMZ + RT. The primary endpoint was overall survival (OS). The planned sample size was 120 (one-sided alpha 0.2; power 0.8).

**Results:**

Between Apr 2010 and Jan 2012, 122 patients were randomized. The median OS with TMZ + RT and TMZ + IFNβ + RT was 20.3 and 24.0 months (HR 1.00, 95% CI 0.65–1.55; one-sided log rank P = 0.51). The median progression-free survival times were 10.1 and 8.5 months (HR 1.25, 95% CI 0.85–1.84). The incidence of neutropenia with the TMZ + RT and the TMZ + IFNβ + RT (grade 3–4, CTCAE version 3.0) was 12.7 versus 20.7% during concomitant period and was 3.6 versus 9.3% during maintenance period. The incidence of lymphopenia was 54.0 versus 63.8% and 34.5 versus 41.9%.

**Conclusions:**

TMZ + IFNβ + RT is not considered as a candidate for the following phase III trial, and TMZ + RT remained to be a most promising treatment. This trial was registered with the UMIN Clinical Trials Registry: UMIN000003466.

## Introduction

Gliomas account for approximately 40% of all brain tumors and are thus the most common primary tumors of the central nervous system (CNS) [1]. In particular, glioblastoma (GBM) is one of the most frequent brain tumors in the CNS in adults and is highly malignant, with a median survival time of about 1 year from diagnosis [2]. An international randomized trial by the European Organisation for Research and Treatment of Cancer (EORTC)/National Cancer Institute of Canada that compared concomitant radiotherapy (RT) and temozolomide (TMZ) to RT alone clearly demonstrated the benefits of adjuvant TMZ chemotherapy for GBM patients [3]. The median OS in the GBM patients who received RT + TMZ in trials in Europe [3], the United States [4], and an international collaboration (AVAglio) [5] were 14.6, 16.8, and 15.7 months, respectively.

Since then, TMZ has been the current first-line chemotherapeutic agent for GBM. A subgroup analysis in the trial above revealed the effectiveness of epigenetic silencing of the *O*^6^-methylguanine-DNA methyltransferase (*MGMT*) gene via promoter methylation, with longer survival, in patients with primary GBM. It also suggested the benefits of agents targeting *MGMT* combining with TMZ plus radiotherapy [6]. Interferonβ (IFNβ) exerts pleiotropic biological effects [7, 8] and has been widely used either as a single agent or in combination with other antitumor agents in the treatment of malignant gliomas and melanomas [9]. In the treatment of malignant gliomas, IFNβ can act as a drug sensitizer, and it enhances the toxicity of chemotherapeutic agents against various neoplasms when administered in combination with nitrosourea [10]. Combination therapy with IFNβ and nitrosourea has been used primarily in the treatment of gliomas in Japan [11]. In our previous in vitro study of human glioma cells, we found that IFNβ markedly enhanced chemosensitivity to TMZ [12]. This finding suggested that one of the major mechanisms by which IFNβ enhances chemosensitivity is the downregulation of *MGMT* transcription via p53 induction. This effect was also observed in an experimental animal model [13]. The results of these 2 studies suggested that chemotherapy with IFNβ and TMZ with concomitant RT might further improve the clinical outcome of patients with malignant gliomas, comparing to chemotherapy with TMZ alone and concomitant RT. Based on these results, we translated the preclinical evidence to clinical studies. A phase I study showed the safety and feasibility of chemotherapy with IFNβ and TMZ combined with concomitant radiotherapy [14, 15]. In addition, a retrospective study demonstrated that addition of IFNβ for newly diagnosed primary GBM achieved a favorable outcome, particularly in patients with an unmethylated *MGMT* promoter [16].

Based on the rationale shown above, we conducted a randomized screening phase II trial of chemoradiotherapy with TMZ plus IFNβ in comparison with chemoradiotherapy with TMZ alone for newly diagnosed GBM (JCOG0911 INTEGRA study), as the Japan Clinical Oncology Group (JCOG) study to explore the superiority of TMZ + IFNβ therapy to TMZ alone in terms of overall survival (OS) in patients with newly diagnosed GBM.

## Materials and methods

### Patients

For inclusion in the study, patients had to meet all of the following criteria: histologically proven newly diagnosed GBM based upon WHO 2007 (IARC 4th edition); 50% of the tumor located in supratentorial areas, without involvement of the optic, olfactory nerves, and pituitary gland and without multiple, disseminated, or large tumors in which the planned irradiated target volume exceeds one-third of the whole brain volume; enrollment 3–20 days after surgery; age between 20 and 75 years; Eastern Cooperative Oncology Group (ECOG) performance status of 0–2 or 3 (only if caused by the tumor); no history of previous chemotherapy or radiotherapy; appropriate organ function and written informed consent.

### Treatment

Patients in the TMZ + RT arm received RT (2.0 Gy/fr/day, 30 fr) with TMZ (75 mg/m^2^, daily) followed by maintenance of TMZ (100–200 mg/m^2^/day, days 1–5, every 4 weeks) for 2 years because (1) optimal duration of maintenance temozolomide had not been determined, and (2) the majority of the investigators in this study agreed that the maintenance temozolomide period was > 12 months.

**Fig. 1:**
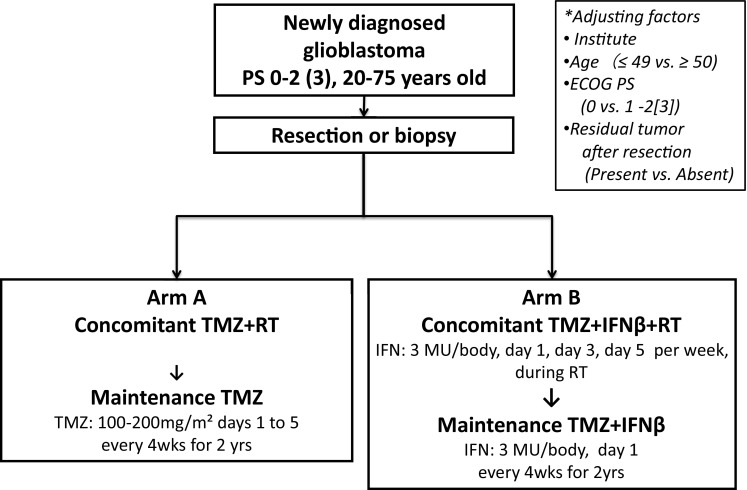
Patient flow diagram of a randomized screening phase II trial of chemoradiotherapy with interferonβ plus temozolomide in comparison with chemoradiotherapy with temozolomide alone for newly diagnosed glioblastoma

Patients in the TMZ + IFNβ + RT arm intravenously received IFNβ (3 MU/body on day 1, day 3, and day 5 during RT concomitant period and day 1, every 4 weeks during the maintenance period) in addition to TMZ + RT (Fig. 1). We determined IFN-beta dosage based on previously published trials, including a Phase I trial [11, 14, 17–20].

RT with concomitant chemotherapy was started within 3 weeks after the surgery. Three-dimensional conformal radiotherapy was planed. Quality assurance reviews were performed at the Radiotherapy Support Center under supervision of JCOG Radiotherapy Committee, with feedback sent to each institution by the RT study coordinator (Minako Sumi). The minimum and maximum doses in the planning target volume (PTV) should comprise between 90 and 110% of the reference point dose of the International Commission on Radiation Units. Gross tumor volume (GTV) was defined as residual tumor, with or without enhancement on computed tomography or magnetic resonance imaging. The clinical target volume 1 (CTV1) included GTV, the resection cavity, and surrounding edema (high-intensity area on T2-weighted or fluid-attenuated inversion recovery image) plus a 1.5-cm margin. The CTV2 included GTV and the resection cavity plus a 1.5-cm margin. PTV was defined as CTV plus a margin of 0.5 cm. The doses for PTV1 and PTV2 were 50 Gy in 25 fractions and 10 Gy in 5 fractions, respectively.

### Study design

This trial was designed as a multicenter, prospective, randomized screening phase II study to explore the superiority of TMZ + IFNβ therapy to TMZ alone in terms of OS in patients with newly diagnosed GBM and to decide whether TMZ + IFNβ should be evaluated in a succeeding confirmatory phase III trial. Patients were randomized using a minimization method with biased-coin assignment to receive either the standard arm (TMZ + RT) or the experimental arm (TMZ + IFNβ + RT) at the JCOG Data Center, adjusting for factors including institution, age (≤ 49 vs. ≥ 50 years), ECOG performance status (0 vs. 1 or 2 [3 if this was due to brain tumor]), and residual tumor after resection (present vs. absent). The study protocol was approved by the JCOG Protocol Review Committee and the institutional review board of each participating institution, and carried out in accordance with the Declaration of Helsinki. This trial was registered at the UMIN Clinical Trials Registry as UMIN000003466 (http://www.umin.ac.jp/ctr/index.htm).

### Statistical consideration

The primary endpoint was OS. OS was calculated from the date of randomization until death from any cause. The secondary endpoints were progression-free survival (PFS), complete response rate, overall response rate, and adverse events. PFS was calculated from the date of randomization until the date of documented progression or death. Responses were evaluated according to Response Evaluation Criteria in Solid Tumors version 1.0. Toxicities were evaluated according to the Common Terminology Criteria for Adverse Events (CTCAE) version 3.0.

The planned sample size was 120 and the expected number of events was 70, with a one-sided alpha of 0.2 and power of 0.8 to detect a difference between arms. The 1-year survival was presumed to be 65% in the TMZ + RT arm, and was expected to be 75% in the TMZ + IFNβ + RT arm. The planned accrual and follow-up period were 1.5 and 2 years, respectively. Primary analysis was conducted 2 year after the accrual completion.

One interim analysis was scheduled after the half of the planned sample size was enrolled to assess the futility of this study. Multiplicity was not taken into consideration because terminating the trial due to superiority of TMZ + IFNβ + RT arm was not planned. Results of interim analysis were reviewed by the JCOG Data and Safety Monitoring Committee and investigators were masked to the results.

OS was analyzed by the stratified log-rank test with residual tumor after resection (present vs. absent) as a strata. Hazard ratio was estimated by stratified Cox proportional hazard model with residual tumor after resection (present vs. absent) as a strata. PFS was analyzed by the unstratified log-rank test and unstratified Cox proportional hazard model. OS and PFS curves were estimated by the Kaplan–Meier method. The efficacy analyses were by intention-to treat and safety analyses were by all patients who received protocol treatment. All analyses were performed by the JCOG Data Center using SAS 9.2 (SAS Institute, Cary, NC).

## Results

### CONSORT diagram and characteristics of the ITT population

**Fig. 2:**
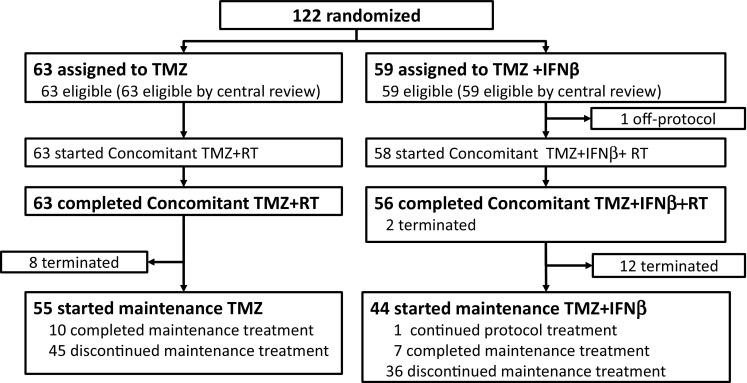
Consort diagram. From April 2010 to January 2012, 122 patients were accrued, of whom 63 and 59 patients were assigned to the TMZ + RT and TMZ + IFNβ + RT arms, respectively

From April 2010 to January 2012, 122 patients were accrued, of whom 63 and 59 patients were assigned to the TMZ + RT and TMZ + IFNβ + RT arms, respectively (Fig. 2). All the tumors were proven to be GBM by the central pathological review. In addition, IDH1/2 mutation in each tumor was not detected though anti-IDH1-R132H immunohistochemistry and Sanger sequencing (Table 1). The patients’ characteristics were as follows: median age (61 years [range 22–75 years] vs. 61 years [range 30–73 years]), male/female (38/25 vs. 35/24), ECOG performance status 0/1–3 (16/47 vs. 12/47), residual tumor resection absent/present (31/32 vs. 33/26) (Table 1). One patient in the TMZ + IFNβ + RT arm was off-protocol before the initiation of protocol treatment owing to liver dysfunction and thus was excluded from the safety analysis. Two patients in the TMZ + IFNβ + RT arm terminated protocol treatment during the concomitant period due to progression and adverse events (grade 3 anorexia and grade 2 erythema multiforme) and 12 patients terminated protocol treatment due to progression, adverse events and patient refusal with adverse events (7, 3, and 2 patients) in the interval between the concomitant and the maintenance treatments. In the TMZ + RT arm, 8 patients terminated protocol treatment during the interval between the concomitant and the maintenance treatments because of progression or adverse events (7 and 1 patients). In the maintenance period, 55 patients started TMZ, but 45 patients terminated the maintenance treatment owing to progression, adverse events, and patient refusal with adverse events (34, 3, and 6 patients) in the TMZ + RT arm. In the TMZ + IFNβ + RT, 44 patients started TMZ + IFNβ, but 36 patients terminated the maintenance treatment owing to progression, adverse events, patient refusal with adverse events, and another reason (30, 1, 3, and 1 patients) (Table 2). One treatment related death (TRD) was observed in TMZ + IFNβ + RT arm during the maintenance therapy (severe renal failure). The post-protocol treatments are listed in Table 3. Chemotherapy using either TMZ or TMZ + IFNβ was administered as post-protocol treatments in 11 and 14 patients, respectively. Other chemotherapies were applied in 1 and 6 patients. Bevacizumab was used in 3 patients in the TMZ + RT arm.

**Table 1:** Characteristics of the ITT population

	TMZ + RT (n = 63)	TMZ + IFNβ + RT (n = 59)
Age, median (range)	61 (22–75)	61 (30–73)
Gender		
Male	38	35
Female	25	24
ECOG PS		
0	16	12
1–3	47	47
Residual tumor after resection		
Absent	31	33
Present	32	26
IDH1/2 status		
No mutation	57	58
Not examined	6	1

**Table 2:** Number of courses of the maintenance treatments

Number of treatment course	TMZ + RT (n = 63)	TMZ + IFNβ + RT (n = 56)	n = 119
0	8(12.7%)	12(21.4%)	20
1–12	39(61.9%)	29(51.8%)	68
13–31	16(25.4%)	15(26.8%)	31

**Table 3:** Post-protocol treatments

	RT/TMZ (n = 39)	RT/TMZ/IFNβ (n = 39)
1. Same as protocol treatment	11	14
TMZ	9	5
TMZ + IFNβ	2	9
2. Other chemotherapy (ACNU, Irinotecan, ICE, other TMZ regimens)	1	6
3.SRS, SRT	4	7
4. Surgery	18	11
5. Others	5	1
Bevacizumab	3	0
Vaccine	2	1

### Overall and progression-free survival

**Fig. 3:**
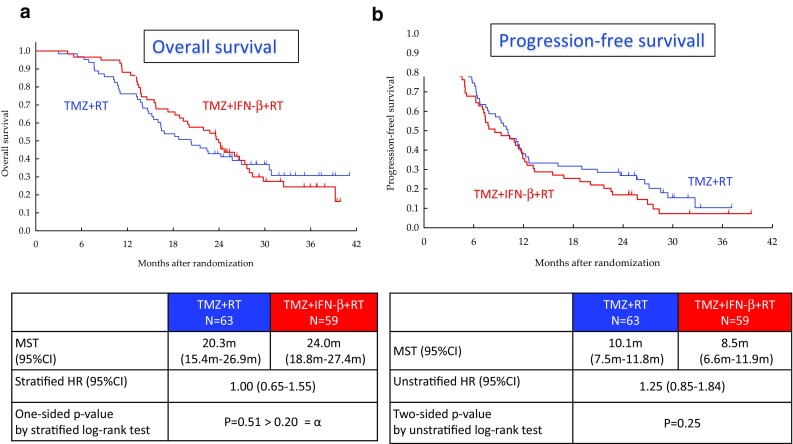
Clinical outcomes. The median survival time was 20.3 months (95% CI 15.4–26.9 months) and 24.0 months (95% CI 18.8–27.4 months) in the TMZ + RT arm and the TMZ + IFNβ + RT arm, respectively (HR 1.00, 95% CI 0.65–1.55; one-sided log rank P = 0.51). OS did not statistically differ between the two arms (**a**). The median PFS was 10.1 months (95% CI 7.5–11.8 months) and 8.5 months (95% CI 6.6–11.9 months) in the TMZ + RT arm and the TMZ + IFNβ + RT arm, respectively (HR 1.25, 95% CI 0.85–1.84; two-sided P = 0.25) (**b**)

The median survival time was 20.3 months (95% CI 15.4–26.9 months) and 24.0 months (95% CI 18.8–27.4 months) in the TMZ + RT arm and the TMZ + IFNβ + RT arm, respectively (HR 1.00, 95% CI 0.65–1.55; one-sided log rank P = 0.51). OS did not statistically differ between the two arms (Fig. 3a).

The median PFS was 10.1 months (95% CI 7.5–11.8 months) and 8.5 months (95% CI 6.6–11.9 months) in the TMZ + RT arm and the TMZ + IFNβ + RT arm, respectively (HR 1.25, 95% CI 0.85–1.84; two-sided P = 0.25) (Fig. 3b).

**Fig. 4:**
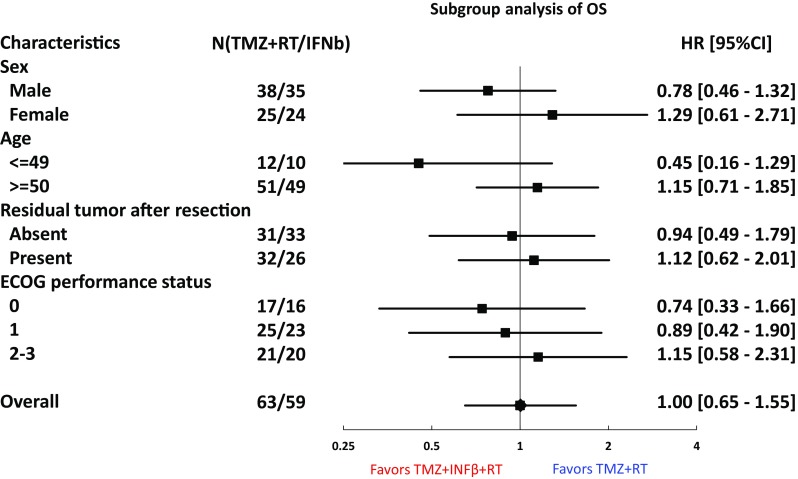
Subgroup analyses were performed for OS by sex (male/female), age (≤ 49 years/≥ 50 years), residual tumor after resection (absent/present) and ECOG PS (0/1/2–3). Male, Younger patients (≤ 49 years) and ECOG PS 0 in the TMZ + IFNβ + RT arm showed good OS compared with RT/TMZ arm

Subgroup analyses were performed for OS by sex (male/female), age (≤ 49 years/≥ 50 years), residual tumor after resection (absent/present) and ECOG PS (0/1/2–3) (Fig. 4). Male, Younger patients (≤ 49 years) and ECOG PS 0 in the TMZ + IFNβ + RT arm showed good OS compared with RT/TMZ arm.

### Adverse events

The incidence of grade 3 and 4 neutropenia was higher in the TMZ + IFNβ + RT arm (Table 4). The difference was more marked in the patients aged ≥ 50 years. Among the non-hematological adverse events, fever, nausea/vomiting, and appetite loss tended to be more frequent in the TMZ + IFNβ + RT arm.

**Table 4:** (a) Adverse events (concomitant chemoradiotherapy), (b) adverse events (maintenance therapy)

(a)	TMZ + RT (N = 63)			TMZ + IFNβ + RT (N = 58)		
	Grade 1–2 (%)	Grade 3 (%)	Grade 4 (%)	Grade 1–2 (%)	Grade 3 (%)	Grade 4 (%)
Hematological						
Neutropenia	25 (39.7)	4 (6.3)	4 (6.3)	32 (55.2)	10 (17.2)	2 (3.5)
Lymphopenia	24 (38.1)	28 (44.4)	6 (9.5)	20 (34.5)	30 (51.7)	7 (12.1)
Non-hematological						
Fever	12 (19.0)	0	0	18 (31.0)	1 (1.7)	0
Nausea	18 (28.6)	0	0	18 (31.0)	2 (3.4)	0
Vomiting	7 (11.1)	0	0	10 (17.2)	1 (1.7)	0
Anorexia	26 (41.3)	0	0	26 (44.8)	5 (8.6)	0
Febrile neutropenia	–	1 (1.6)	0	–	2 (3.4)	0
ALT elevation	35 (55.6)	6 (9.5)	0	31 (53.4)	5 (8.6)	1 (1.7)
Hyponatremia	13 (20.6)	3 (4.8)	0	15 (25.9)	5 (8.6)	0
Skin rash	13 (20.6)	1 (1.6)	0	4 (6.9)	0	0

(b)	TMZ + RT (N = 55)			TMZ + IFNβ + RT (N = 43)^a^		
	Grade 1–2 (%)	Grade 3 (%)	Grade 4 (%)	Grade 1–2 (%)	Grade 3 (%)	Grade 4 (%)
Hematological						
Neutropenia	31 (56.4)	2 (3.6)	0	21 (48.8)	4 (9.3)	0
Lymphopenia	29 (52.7)	17 (30.9)	2 (3.6)	22 (51.2)	17 (39.5)	1 (2.3)
Non-hematological						
Fever	3 (5.5)	0	0	5 (11.6)	0	0
Nausea	14 (25.5)	0	0	6 (14.0)	0	0
Vomiting	4 (7.3)	1 (1.8)	0	2 (4.7)	0	0
Anorexia	16 (29.1)	2 (3.6)	0	7 (16.3)	0	0
Febrile neutropenia	–	0	0	–	0	0
ALT elevation	30 (54.5)	1 (1.8)	0	20 (46.5)	0	0
Hyponatremia	11 (20.0)	0	0	7 (16.3)	2 (4.7)	0
Skin rash	8 (14.5)	0	0	8 (18.6)	0	0

According to the Common Terminology Criteria for Adverse Events (CTCAE) version 3.0

^a^Data of one patient is missing

## Discussion

IFNs exert pleiotropic antitumor effects by direct anticancer mechanisms though p53 induction and miR-21 downregulation or by regulating the immune system through the CD8 lymphocyte and macrophage activation [7]. This study was a randomized screening phase II trial to explore the superiority of TMZ + IFNβ therapy to TMZ alone for the patients with newly diagnosed GBM. In the present study, the superiority of TMZ + IFNβ + RT to TMZ + RT in OS was not demonstrated.

There are some possibilities that we failed to show the superiority of TMZ + IFNβ + RT to TMZ + RT. One potential reason is that TMZ + IFNβ + RT treatment was more toxic than expected. Before we started this trial, we assumed that additional IFNβ would not increase much toxicity because it had been suggested in some reports using nitrosourea anti-tumor agent with IFNβ. However, the proportion of severe (grade 3–4) hematological and non-hematological adverse events was higher in the TMZ + IFNβ + RT arm than in the TMZ + RT arm, which implied such unexpected severe toxicities could cause negative impact on the survival in the TMZ + IFNβ + RT arm. Due to the severe toxicities, treatment compliance was also deteriorated in the TMZ + IFNβ + RT arm. In fact, the number of patients who terminated protocol treatment before the start of the maintenance treatments was larger in the TMZ + IFNβ + RT arm, which was possibly caused by the toxicities as mentioned above. *MGMT* methylation status has not been investigated yet, but we are planning to evaluate the biomarkers including *MGMT* gene expression and methylation using tumor tissues and blood samples.

Subgroup analyses showed IFNβ could be possibly beneficial for younger, male, better PS, no residual tumor patients. It may suggest that the better tolerability against IFNβ toxicities might be predictive factors of IFNβ efficacy, but further studies would be needed to confirm this hypothesis.

As the future direction, we will seek for the promising combination therapy with TMZ + RT and other agents than IFNβ as a candidate of the following study for GBM. Now we just started a randomized phase III trial to confirm the superiority of dose-dense TMZ (ddTMZ) followed by bevacizumab at ddTMZ failure to bevacizumab alone for patients with first recurrence or progression of GBM (JCOG1308C).

In conclusion, although the combination therapy of TMZ + IFNβ + RT showed favorable survival, the superiority of TMZ/IFNβ + RT to TMZ + RT in overall survival was not demonstrated. Therefore TMZ + IFNβ + RT was not considered promising as the test treatment in the following phase III study for newly diagnosed GBM and TMZ + RT remained to be a most promising treatment.

## Abbreviations

TMZ: Temozolomide
IFNβ: Interferonβ
RT: Radiotherapy
OS: Overall survival
MU: Million unit
GBM: Glioblastoma
MGMT: *O*^6^-methylguanine-DNA methyltransferase
PTV: Planning target volume
GTV: Gross tumor volume
CTV: Clinical target volume
PFS: Progression-free survival
CTCAE: Common terminology criteria for adverse events
ACNU: Nimustine hydrochloride
HR: Hazard ratio
JNS: Japan Neurosurgical Society
IARC: International Agency for Research on Cancer
